# Endovascular treatment of aortic saccular aneurysms associated with Adamantiades-Behçet disease

**DOI:** 10.1590/1677-5449.200201

**Published:** 2021-06-25

**Authors:** Patrick Bastos Metzger, Kamilla Rosales Costa, Simone Lessa Metzger, Leonardo Cortizo de Almeida

**Affiliations:** 1 Escola Bahiana de Medicina e Saúde Pública – EBMSP, Salvador, BA, Brasil.; 2 Universidade Salvador – UNIFACS, Salvador, BA, Brasil.; 3 Obra Sociais Irmã Dulce – OSID, Hospital Santo Antônio, Salvador, BA, Brasil.; 4 Hospital Geral Roberto Santos – HGRS, Salvador, BA, Brasil.

**Keywords:** Behçet syndrome, aortic aneurysm, endovascular procedures

## Abstract

Adamantiades-Behçet disease is a multisystemic disorder that classically presents with oral and genital ulcers and ocular involvement, with vascular involvement in up to 38% of cases. Aortic involvement is one of the most serious manifestations and is associated with high mortality rates, occurring in 1.5 to 2.7% of cases. We report a case of a saccular abdominal aorta aneurysm in a 49-year-old male patient with complicated Adamantiades-Behçet disease that was treated with endovascular repair.

## INTRODUCTION

Adamantiades-Behçet disease (ABD) is a rare, inflammatory, multisystemic syndrome with unknown pathophysiology that can involve the gastrointestinal tract, the cardiovascular system, the joints, and the central nervous system, in addition to possible cutaneous manifestations.^1^^,^^2^ Vascular involvement is present in 7 to 38% of ABD cases, most often affecting the venous system, in up to 75% of cases.^2^^,^^3^ The prevalence of arterial lesions reported in the literature is 3.6 to 31%, with presentations that range from aneurysms to acute arterial stenosis or thromboses, most frequently affecting the renal, pulmonary, and abdominal arteries.^2^^,^^3^ Aortic involvement is one of the most serious manifestations and is associated with high mortality rates, occurring in 1.5 to 2.7% of cases and usually presenting as pseudoaneurysms or saccular aneurysms of the abdominal aorta.^1^^,^^3^^,^^4^ Open surgery is considered complicated and technically challenging in these cases.^3^^,^^4^

There is no universally accepted test to diagnose ABD and diagnosis is therefore based on the classic triad established by the International Study Group for Behçet's Disease, comprising oral ulcers, genital ulcers, and uveitis.^1^^,^^4^^,^^5^

We report a case of ABD with arterial and venous involvement, including a complicated saccular abdominal aortic aneurysm (AAA) that was treated via an endovascular approach, and extensive deep venous thrombosis (DVT) secondary to ABD. Approval was granted by the Ethics Committee under decision number 4,466,229. The patient gave his consent to publication of the clinical case and images.

## CASE REPORT

**Figure 1:**
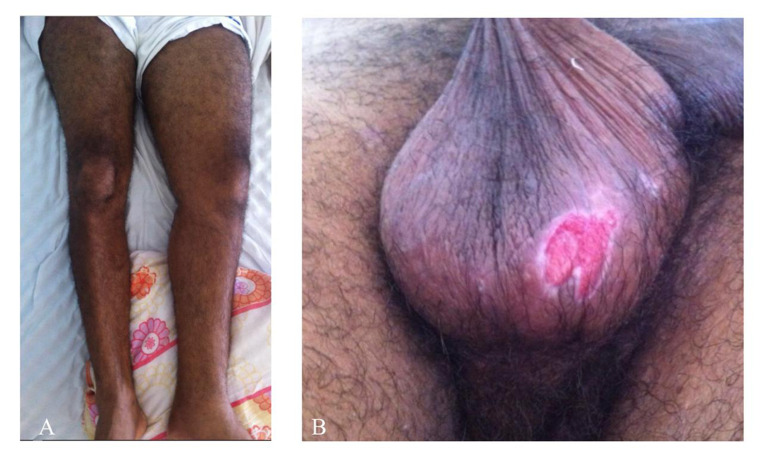
(A) Asymmetrical edema of the left lower limb secondary to iliofemoral deep venous thrombosis; (B) Genital ulcer.

**Figure 2:**
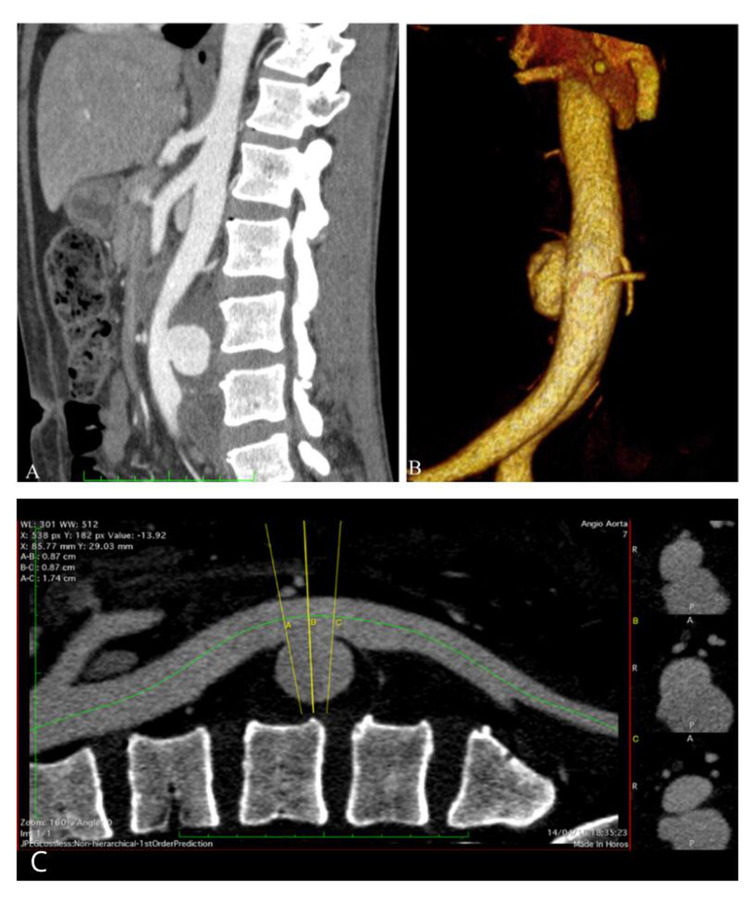
(A) Sagittal image from angiotomography of the abdominal aorta, showing large volume posterior saccular aneurysm; (B) Volumetric angiotomography reconstruction, showing large volume posterior saccular aneurysm; (C) Sagittal angiotomography image of the abdominal aorta, showing aneurysm neck and dimensions.

A 49-year-old man presented with asymmetrical edema of the left lower limb and pain in the ipsilateral thigh and aqueous diarrhea for 3 days prior to hospital admission (Figure 1A). Venous ultrasonography with Doppler of the lower limbs confirmed DVT in the left femoropopliteal venous segment. Full angiotomography of the abdomen was then conducted to assess the extent of iliocaval thrombosis. This examination detected an incidental finding of a posterior saccular aneurysm of the abdominal aorta, close to the L3 vertebral body, predominantly on the left and with eccentric thrombi. The largest diameter was 6.4 cm and the neck measured 1.7 cm (Figure 2). There was also thrombosis of the entire left common and external iliac veins. The patient reported a history of recurrent oral and genital ulcers and arthritis for the pervious 8 years, in addition to acneiform eruptions on his back and face (Figure 1B). He had no history of diabetes mellitus or systemic arterial hypertension and denied consumption of alcohol, tobacco, or other drugs. His erythrocyte sedimentation rate was 64 mm/h, C-reactive protein was 29 mg/L, and D-dimer was 2,558 ng/mL. His total and differential white blood cell counts were normal. Angiotomography of the thorax was conducted and ruled out thoracic aneurysmal involvement and pulmonary embolism.

In view of the clinical status described above, the suspected diagnosis was ABD according to the criteria established by the International Study Group for Behçet's Disease (Table 1) and care was managed in conjunction with the rheumatology team. Initially, the patient was started on treatment with corticosteroids for ABD and enoxaparin 1 mg/kg twice a day for the acute DVT. On his third day in hospital, he developed abdominal and lumbar pain on the left, with no drop in hemoglobin levels and hemodynamic stability maintained. Another angiotomography examination was conducted, showing that the aneurysm had expanded to 7.8 cm with posterior infiltration, close to the vertebrae. The decision was taken to withdraw enoxaparin and administer fresh plasma, implant an infrarenal inferior vena cava filter via the left femoral vein, and perform endovascular repair of the AAA.

**Table 1:** International Study Group for Behçet's Disease diagnostic criteria*

**Primary criterion:**
• **Recurrent oral ulcers; at least 3 times in a 1-year period.**
**Secondary criteria:**
• **Recurrent aphthous genital ulcers**
• **Ocular:**
Anterior and/or posterior uveitis.
Retinal vasculitis.
• **Cutaneous manifestations:**
Erythema nodosum, pseudofolliculitis, acneiform papulopustular lesions outside of puberty and without corticoids.
Positive pathergy test.

Diagnosis is confirmed if the primary criterion and two secondary criteria are present.

* Criteria from International Study Group for Behçet's Disease^5^

**Figure 3:**
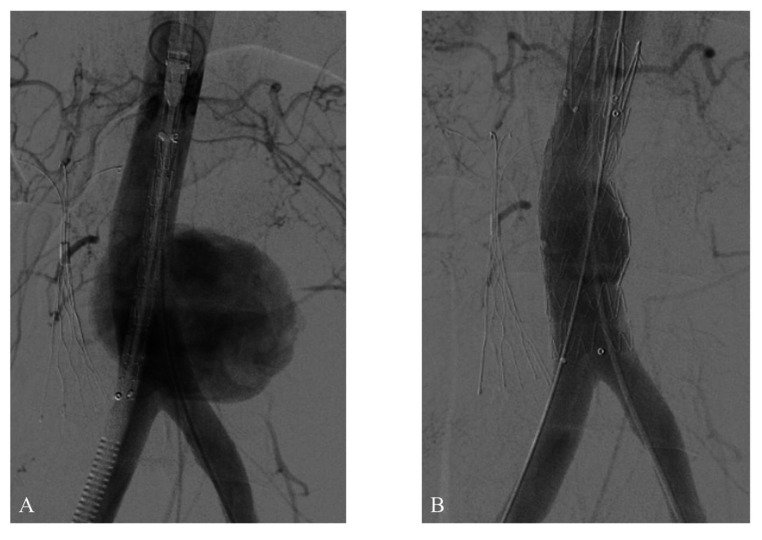
(A) Anteroposterior abdominal aortography before deployment, the large volume saccular aneurysm; (B) Control angiography after deployment of the endograft, with no leaks.

In the interventional radiology suite, a 22 × 22 × 100 mm straight Valiant® stent graft (Medtronic, Santa Rosa, California, United States) was deployed into the infrarenal abdominal aorta via open dissection of the right femoral artery, preserving the aortic bifurcation and occluding the neck of the aneurysm. Control aortography showed the graft was correctly positioned and free from leaks (Figures 3A and 3B).

Postoperatively, the patient was transferred to the intensive care unit (ICU), where he recovered well, with no elevation of nitrogenous wastes and with all lower limb pulses present. He was discharged from the ICU on the second postoperative day and from hospital on the seventh day, with reintroduction of corticosteroids at an ambulatory level. At 2 years' follow-up, the patient's good postoperative results are maintained. Control tomography examinations show no signs of leaks, he is free from symptoms of ABD, and is taking an immunosuppressant (azathioprine).

## DISCUSSION

Adamantiades-Behçet disease is a rare syndrome that has greater prevalence in areas along the ancient silk trade route, in the Middle East and Central Asia, and in Japan. If affects both sexes, but has greater severity in males. It predominantly affects people aged from 15 to 45 years and peak incidence is at 30 years of age.^1^^,^^3^^,^^5^^,^^6^ Although its etiology has not been fully explained, studies demonstrate associations with HLA-B51 and HLA-B27 antigens.^1^ There are studies investigating other factors related to the pathophysiology of the disease, such as family history and microbial infections including the herpes simplex virus and *Streptococcus sanguis* bacteria.^7^

Clinical status is characterized by acute and recurrent inflammatory episodes, with oral and genital aphthous ulcers, uveitis, and cutaneous lesions, which are common and self-limiting manifestations, with the exception of uveitis, which can manifest a severe and progressive course and can even progress to blindness.^1^ Since there is no laboratory test to diagnose ABD, suspicion is based on the disease's classic triad: oral ulcers, genital ulcers, and uveitis.^1^^,^^4^ The International Study Group for Behçet's Disease^5^ has established diagnostic criteria for ABD, based on signs and symptoms (Table 1).

Vasculitis is the most common clinical event among the disease's clinical vascular manifestations.^1^ The constant inflammatory process causes progressive destruction of elastic and muscular cells in the artery wall. Occlusion of the vasa vasorum contributes to this process through transmural necrosis, with later perforation and pseudoaneurysm formation.^3^ Aneurysms can involve any arterial territory, although abdominal, femoral, and pulmonary arteries are the most commonly involved.^7^ During aneurysm formation, the patient may present with strong pain caused by the inflammatory process, but diagnosis of an AAA demands a high index of suspicion.^3^ This manifestation is most common among men and mean time of development is in the range of 5 to 9 years after diagnostic criteria for ABD are established.^1^^,^^3^^,^^8^ Although aneurysms are rare in ABD, rupture is the most common cause of death in this disease, justifying treatment as soon as they aneurysms are identified.^6^^,^^7^ Survival rates after diagnosis of arterial involvement are 83% at 5 years and 66% at 15 years.^6^ Aneurysm rupture is caused by the inflammatory process and fibrotic reactions in tissues surrounding the aneurysm and there is a risk of rupture regardless of the diameter of the vessel.^6^^,^^9^ Aneurysmal lesions in patients with ABD should therefore be treated.^1^^,^^4^^,^^6^^,^^8^^,^^9^

There are three treatment possibilities for aneurysms secondary to ABD: clinical, open surgical, or via endovascular approaches. Traditionally, clinical treatment consists of administration of corticoid and immunosuppressants (cyclophosphamide and azathioprine), but does not however reduce mortality due to rupture or occlusion of vessels when used in isolation. Recent studies report reduction of corticoid dosages and relapse rates when the corticoid is combined with anti-TNFα, resulting in regression of the aneurysm in 3 months. Clinical treatment can be employed during the period preceding surgical treatment and shows improved results. Surgical repair of aortic aneurysms can be accomplished in two different ways: via resection and substitution with a prosthetic graft, reinforced or not with a free omentum patch,^6^^,^^10^ or by aneurysmectomy with direct closure using a pericardium patch for saccular aneurysms.^6^^,^^11^ However, this type of approach can complicate in around 30 to 50% of cases, particularly when performed during the active phase of the disease.^1^^,^^3^^,^^6^^,^^7^^,^^12^^,^^13^

Since 1984, a growing number of investigators have reported the efficacy of endovascular treatment for AAA in patients with ABD. Regular monitoring of these patients can detect possible complications, such as femoral pseudoaneurysms (at puncture sites), migrations, fractured devices, and late leaks.^14^

Park et al.^14^ described treatment of three patients with ABD and aortic aneurysms (thoracic, suprarenal, and infrarenal) using endovascular repair. In the patient with the infrarenal aorta aneurysm, the aneurysm recurred below the endoprosthesis and was treated with a second endovascular repair procedure. The other two patients had no complications. Follow-up duration was 31, 40, and 59 months, respectively.

Nitecki et al.,^15^ compared the results of surgical treatment with endovascular repair in five patients with ABD treated for infrarenal AAA, demonstrating that the patients treated with open surgery had higher morbidity and mortality rates than the group treated with endovascular repair (one death, one major amputation, and three pseudoaneurysms vs. one episode of transitory acute renal failure).

In the case reported here, it was not possible to employ a bifurcated endograft because there were none available that would fit the small aortic and iliac diameters and the case was an emergency. This is why the decision was taken to use a small diameter straight thoracic stent graft that was available at the time to treat the saccular infrarenal abdominal aorta aneurysm, with a good final result.

Use of thoracic stent grafts in the aortoiliac segment has been described in the literature as a reasonable alternative option for treatment of complex infrarenal aortic aneurysms, in which use of the chimney technique or branched or fenestrated endografts may be necessary. However, greater experience with the technique (Funnel Technique) and longer follow-up are needed to define use of this material.^16^^,^^17^ Undoubtedly, fitting a straight thoracic endograft would not have been our first choice if low profile 2-component or 3-component stent-graft systems with appropriate diameters had been available. Additionally, in a patient with active ABD, open surgery would have been high risk and may have had an unfavorable outcome.

We conclude that endovascular treatment of an AAA complicated by ABD is effective. The result in this case was satisfactory over a medium-term follow-up period.
