# Supplementary material for: Endovascular treatment of aortic saccular aneurysms associated with Adamantiades-Behçet disease
Source: J Vasc Bras. 2021 Jun 25;20:e20200201. doi: 10.1590/1677-5449.200201 (PMC8244983; doi:10.1590/1677-5449.200201)
Supplement: Translation

# Translation: Tratamento endovascular de aneurisma sacular aórtico associado à doença de Adamantiades-Behçet

**Authors:** Patrick Bastos Metzger, Kamilla Rosales Costa, Simone Lessa Metzger, Leonardo Cortizo de Almeida

**Affiliations:** 1 Escola Bahiana de Medicina e Saúde Pública – EBMSP, Salvador, BA, Brasil.; 2 Universidade Salvador – UNIFACS, Salvador, BA, Brasil.; 3 Obra Sociais Irmã Dulce – OSID, Hospital Santo Antônio, Salvador, BA, Brasil.; 4 Hospital Geral Roberto Santos – HGRS, Salvador, BA, Brasil.

**Keywords:** síndrome de Behçet, aneurisma aórtico, procedimentos endovasculares

## Abstract

A doença de Adamantiades-Behçet é uma desordem multissistêmica que se apresenta classicamente com úlceras orais e genitais e envolvimento ocular, podendo o acometimento vascular ocorrer em até 38% dos casos. O envolvimento aórtico é uma das manifestações mais severas e está associado a altas taxas de mortalidade, ocorrendo em 1,5 a 2,7% dos casos. Relatamos um caso de aneurisma sacular de aorta abdominal em um paciente de 49 anos com doença de Adamantiades-Behçet complicada, tratada por correção endovascular.

## INTRODUÇÃO

A doença de Adamantiades-Behçet (DAB) é uma síndrome rara, de caráter inflamatório e multissistêmico, podendo acometer o trato gastrointestinal, o sistema cardiovascular, as articulações e o sistema nervoso central, além de possíveis manifestações cutâneas, sendo sua fisiopatologia ainda desconhecida^1^^,^^2^. O acometimento vascular está presente em cerca de 7 a 38% dos casos de DAB, sendo o sistema venoso o mais afetado em até 75% dos casos^2^^,^^3^. As lesões arteriais têm uma prevalência entre 3,6 a 31% na literatura e variam desde aneurismas até estenoses ou tromboses arteriais agudas, sendo as artérias renais, pulmonares e abdominais as mais afetadas^2^^,^^3^. O envolvimento aórtico é uma das manifestações mais severas e está associado a altas taxas de mortalidade, ocorrendo em 1,5 a 2,7% dos casos, apresentando-se usualmente na forma de pseudoaneurismas ou aneurismas saculares em aorta abdominal^1^^,^^3^^,^^4^. A cirurgia aberta, nesses casos, é considerada complicada e tecnicamente desafiadora^3^^,^^4^.

Não existe nenhum teste universalmente aceito para o diagnóstico de DAB. Assim, ele é realizado através da presença de sua tríade clássica, estabelecida pelo *International Study Group for Behçet's Disease*: úlceras orais, úlceras genitais e uveíte^1^^,^^4^^,^^5^.

Relatamos um caso de acometimento arterial e venoso na DAB, com um aneurisma de aorta abdominal (AAA) sacular complicado, tratado via endovascular, e uma trombose venosa profunda (TVP) extensa, secundários à DAB. Houve aprovação do Comitê de Ética, sob parecer número 4.466.229. O paciente consentiu com a publicação do caso clínico e das imagens.

## RELATO DO CASO

Homem de 49 anos com queixa de edema assimétrico de membro inferior esquerdo associado a dor em coxa ipsilateral e diarreia aquosa por 3 dias antes da admissão hospitalar (Figura 1A). Realizou-se uma ultrassonografia com Doppler venoso dos membros inferiores, que confirmou TVP no segmento venoso fêmoro-poplíteo à esquerda. Foi realizada, então, uma angiotomografia de abdome total, para avaliar extensão trombótica cavo-ilíaca, evidenciando, de forma incidental, um aneurisma sacular posterior de aorta abdominal, próximo ao corpo vertebral de L3 com acometimento predominantemente à esquerda e com trombos excêntricos, tendo o maior diâmetro de 6,4 cm e colo de 1,7 cm (Figura 2), bem como trombose de toda veia ilíaca comum e externa esquerda. Em seus antecedentes patológicos, paciente relatava úlceras orais e genitais recorrentes e artrite nos últimos 8 anos, além do surgimento de lesões acneiformes em dorso e face (Figura 1B). Não apresentava história de diabetes melito ou hipertensão arterial sistêmica e negava uso de álcool, tabaco ou outras drogas. Apresentava velocidade de hemossedimentação de 64 mm/h, proteína C reativa de 29 mg/L e dímero-D de 2.558 ng/mL. Leucograma sem alterações na contagem global ou na diferencial. Foi realizada angiotomografia de tórax, sendo afastados outros acometimentos aneurismáticos torácicos e embolia pulmonar.

**Figura 1:** (A) Edema assimétrico em membro inferior esquerdo secundário à trombose venosa profunda ilíaco-femoral; (B) Úlcera genital.

**Figura 2:** (A) Angiotomografia de aorta abdominal em corte sagital, com volumoso aneurisma sacular posterior; (B) Angiotomografia de reconstrução volumétrica, com volumoso aneurisma sacular posterior; (C) Angiotomografia de aorta abdominal em corte sagital, demonstrando colo e dimensão do aneurisma.

Devido ao quadro clínico descrito, foi feita a suspeita diagnóstica de DAB segundo os critérios estabelecidos pelo *International Study Group for Behçet's Disease* (Tabela 1), com acompanhamento conjunto da equipe de reumatologia, e iniciado o tratamento com corticosteroides para DAB e enoxaparina 1 mg/kg duas vezes ao dia para o tratamento inicial da TVP aguda. No terceiro dia de internação hospitalar, o paciente evoluiu com dor abdominal e lombar à esquerda, sem queda de hemoglobina e mantendo estabilidade hemodinâmica. Nova angiotomografia do abdome demonstrava aumento das dimensões do aneurisma para 7,8 cm com infiltração para posterior, próximo das vértebras. Optou-se, então, pela suspensão da enoxaparina e administração de plasma fresco com implante de filtro de veia cava inferior infrarrenal via veia femoral esquerda e tratamento endovascular do AAA.

**Tabela 1:** Critérios diagnósticos do *International Study Group* para doença de Behcet*

**Critério primário:**
• **Úlceras orais recorrentes; pelo menos 3 vezes no período de 1 ano.**
**Critérios secundários:**
• **Úlceras genitais aftosas recorrentes**
• **Ocular:**
Uveíte anterior e/ou posterior.
Vasculite retiniana.
• **Manifestações na pele:**
Eritema nodoso, pseudofoliculite, lesões acneiformes pápulo-pustular fora da puberdade e sem uso de corticoide.
Teste de patergia positivo.

Diagnóstico confirmado com o critério primário e dois secundários presentes.

* Critérios da International Study Group for Behçet's Disease^5^.

O implante da endoprótese Valiant® (Medtronic, Santa Rosa, Califórnia, EUA) reta 22 × 22 × 100 mm foi realizado via dissecção aberta de artéria femoral direita em aorta abdominal infrarrenal, preservando a bifurcação aórtica e com a oclusão do colo aneurismático, em suíte de hemodinâmica. A aortografia de controle demonstrou endoprótese bem posicionada e sem vazamentos (Figuras 3 e 3B).

**Figura 3:** (A) Aortografia abdominal em incidência anteroposterior pré-implante, demonstrando o volumoso aneurisma sacular; (B) Angiografia de controle pós-implante de endoprótese, sem vazamentos.

O pós-operatório foi realizado em unidade de terapia intensiva (UTI), com boa evolução, sem elevação de escórias nitrogenadas e apresentando todos os pulsos dos membros inferiores. O paciente teve alta da UTI no 2º dia de pós-operatório e alta hospitalar no 7º dia, com reintrodução do corticosteroide em nível ambulatorial. Após 2 anos de acompanhamento, o paciente mantém-se com boa evolução operatória, mantendo tomografias de controle sem sinais de vazamentos, sem sintomas da DAB e em uso de imunossupressor (azatioprina).

## DISCUSSÃO

A DAB é uma síndrome rara, mais prevalente nas áreas ao redor da antiga rota do comércio da seda, no Oriente Médio e Ásia Central, e no Japão, acometendo ambos os sexos, porém com maior severidade no sexo masculino. Acomete prioritariamente a faixa etária entre 15 e 45 anos, com pico de incidência aos 30 anos^1^^,^^3^^,^^5^^,^^6^. Apesar de não apresentar etiologia bem esclarecida, estudos demonstram associação com os antígenos HLA-B51 e HLA-B27^1^. Outros fatores estão sendo estudados quanto à relação com a fisiopatologia da doença, como história familiar e infecções microbianas, tais como o vírus herpes simples e a bactéria *Streptococcus sanguis* 7.

O quadro clínico é caracterizado por episódios inflamatórios agudos e recorrentes, marcados por úlceras aftosas orais e genitais, uveíte e lesões cutâneas, manifestações que são comuns e autolimitadas, com exceção da uveíte, que pode evoluir de forma grave e progressiva até a cegueira^1^. Devido à carência de exames complementares para o diagnóstico da DAB, sua suspeita se dá baseada na tríade clássica da doença: úlceras orais, úlceras genitais e uveíte^1^^,^^4^. Os critérios diagnósticos para a DAB, com seus sinais e sintomas, foram estabelecidos pelo *International Study Group for Behçet's Disease* 5 (Tabela 1).

A vasculite é o evento mais comum entre as manifestações clínicas vasculares da doença^1^. Diante do processo inflamatório constante, as células elásticas e musculares da parede arterial são progressivamente destruídas. A oclusão do *vasa vasorum* contribui para esse processo devido à necrose transmural, com posterior perfuração e formação de pseudoaneurisma^3^. O aneurisma pode acometer qualquer território arterial, sendo, no entanto, mais frequente nas artérias do abdome, femorais e pulmonar^7^. Durante a formação aneurismática, o paciente pode se apresentar com dor importante devido ao processo inflamatório instalado, porém o diagnóstico do AAA necessita de alto grau de suspeição^3^. Essa manifestação é mais frequente no sexo masculino e apresenta um tempo médio de desenvolvimento de 5 a 9 anos após o estabelecimento dos critérios diagnósticos da DAB^1^^,^^3^^,^^8^. Apesar dos aneurismas na DAB serem raros, a sua ruptura constitui a principal causa de morte nessa doença, o que justifica o tratamento após a identificação^6^^,^^7^. A taxa de sobrevida após o diagnóstico do acometimento arterial é de 83% em 5 anos e de 66% em 15 anos^6^. A ruptura do aneurisma se dá pelo processo inflamatório dos tecidos perianeurismáticos e das reações fibróticas, e há risco de ruptura independente do diâmetro do vaso^6^^,^^9^. Por essa razão, lesões aneurismáticas em pacientes com DAB devem ser tratadas^1^^,^^4^^,^^6^^,^^8^^,^^9^.

O tratamento do aneurisma secundário à DAB pode ser realizado de três formas: clínico, cirúrgico aberto ou via endovascular. O tratamento clínico consiste tradicionalmente na administração de corticoide e imunossupressores (ciclofosfamida e azatioprina), sem, no entanto, apresentar diminuição da mortalidade quando realizado isoladamente, devido a ruptura ou oclusão dos vasos. Estudos recentes relatam diminuição das doses de corticoide e das taxas de recidiva quando associado o corticoide ao anti-TNFα, com resultados de regressão do aneurisma em 3 meses. A terapêutica clínica pode ser utilizada momentos antes da abordagem cirúrgica e apresenta melhora dos resultados. A correção cirúrgica do aneurisma de aorta pode ser feita de duas formas distintas: por ressecção e substituição com prótese, reforçada ou não por retalho livre do omento^6^^,^^10^, e por aneurismectomia com fechamento direto com uso de *patch* de pericárdio em aneurisma saculares^6^^,^^11^. No entanto, esse tipo de abordagem pode complicar em cerca de 30 a 50% dos casos, principalmente quando realizada na fase ativa da doença^1^^,^^3^^,^^6^^,^^7^^,^^12^^,^^13^.

Desde 1984, um número crescente de investigadores tem relatado a eficácia do tratamento endovascular do AAA em pacientes com DAB. O acompanhamento regular desses pacientes pode detectar possíveis complicações, como pseudoaneurismas femorais (sítios de punção), migrações, fraturas de materiais e vazamentos tardios^14^.

Park et al.^14^ descreveram o tratamento de três pacientes com DAB e aneurismas aórticos (torácico, suprarrenal e infrarrenal) usando o reparo endovascular. Um dos pacientes com aneurisma de aorta infrarrenal teve recorrência do aneurisma abaixo da endoprótese e foi tratado com nova correção endovascular. Os outros dois pacientes não tiveram complicações. A duração do acompanhamento foi de 31, 40 e 59 meses, respectivamente.

Nitecki et al.^15^, comparando os resultados do tratamento cirúrgico com o endovascular em cinco pacientes tratados por AAA infrarrenal com DAB, demonstraram que os pacientes submetidos à cirurgia aberta tiveram maior taxa de morbimortalidade (uma morte, uma amputação maior e três pseudoaneurismas vs. uma insuficiência renal aguda transitória) comparados ao grupo tratado por via endovascular.

Neste relato de caso, não foi possível usar uma endoprótese bifurcada devido à indisponibilidade da mesma no local para um diâmetro aórtico e ilíaco reduzido, em um caso de urgência. Por esse motivo, optou-se pelo uso de uma endoprótese torácica reta de pequeno diâmetro, disponível naquele momento para o tratamento do aneurisma sacular de aorta abdominal infrarrenal, com um bom resultado final.

O uso de entopróteses torácicas no segmento aorto-ilíaco é relatado na literatura como uma alternativa razoável no tratamento de aneurismas aórticos infrarrenais complexos, nos quais o uso da técnica de chaminé, de endopróteses ramificadas ou fenestradas pode ser necessário. Entretanto, maior experiência com essa técnica (*Funnel Technique*) e um tempo de segmento maior são necessários para definir o uso desse material^16^^,^^17^. Sem dúvida, o implante de endoprótese torácica reta não seria nossa primeira escolha, tendo endopróteses bimodulares ou trimodulares de baixo perfil e com diâmetro adequado disponíveis. Além disso, a cirurgia aberta neste paciente com DAB em atividade, além de ser de alto risco, poderia ter um desfecho desfavorável.

Concluímos que o tratamento endovascular do AAA complicado pela DAB é efetivo. O resultado em médio prazo do acompanhamento foi satisfatório neste relato de caso.
